# Decreased angiotensin receptor 1 expression in ± AT1 Knockout mice testis results in male infertility and GnRH reduction

**DOI:** 10.1186/s12958-021-00805-1

**Published:** 2021-08-03

**Authors:** Fangfang Zhao, Yun Zou, Hui Li, Yaheng Zhang, Xuele Liu, Xuehao Zhao, Xinyi Wu, Wenyi Fei, Ziling Xu, Xuejun Yang

**Affiliations:** 1 Institute of Nephrology, Shuguang Hospital, Shanghai University of Traditional Chinese Medicine, 528 Zhangheng Road, Pudong New Area, Shanghai, China; 2 Institute of Nephrology, Guangming Traditional Chinese Medicine Hospital, 339 East gate Street, Pudong New Area, Shanghai, China

**Keywords:** Angiotensin receptor 1, Male infertility, ± AT1KO mice, Testis, Hypothalamic-pituitary–gonadal (HPG) axis

## Abstract

**Background:**

This study aimed to detect the effect of angiotensin receptor 1 (AT1) knock out (KO) on spermatogenesis and hypothalamic-pituitary–gonadal (HPG) axis hormone expression.

**Methods:**

Normal C57BL/6 male mice were used as control group or treated with angiotensin receptor blocker, in addition heterozygous ± AT1KO mice were generated. After caged at a ratio of 2 to 1 with females, pregnancy rates of female mice were determined by detection of vaginal plugs. Deformity rate of spermatozoa was evaluated by eosin staining and morphology evaluation. The AT1 mRNA expression in the testes of male ± AT1KO mice was detected by quantitative real-time polymerase chain reaction (QRT-PCR). Serum GnRH level was determined by ELISA.

**Results:**

Compared to control, ± AT1KO mice showed reduced expression of AT1 in testes, pituitary and hypothalamus. In addition, decreased level of GnRH, but not follicle stimulating hormone (FSH) or luteinizing hormone (LH), in ± AT1KO mice was detected. Treatment with angiotensin receptor blocker (ARB) did not have significant effects on HPG hormones. ± AT1KO mice exhibited male infertility and significant abnormality of sperm morphology.

**Conclusion:**

Reduced AT1 knockout resulted in male infertility, potentially by inducing abnormal spermatogenesis. Both testis and HPG axis signaling may be involved.

## Background

Male infertility is a worldwide problem. Previous studies have shown that male causes accounted for 50% of infertility, in which about 30% of cases are still thought to be idiopathic [1, 2]. In most male fertility cases, abnormal semen parameters, including azoospermia, oligozoospermia, teratozoospermia, asthenozoospermia could be detected. Even in idiopathic normozoospermic male infertility, a dysfunction of spermatozoa maturation in the female reproductive tract is usually the cause [2]. These findings indicate that abnormal spermatogenesis, which causes abnormality include sperm production, quantity, morphology and motility is the main cause of male fertility [2]. Spermatogenesis is a complex process that could be regulated at multiple stages. Although many factors such as genetic, epigenetic, protein expression and post-translational modification, as well as environmental stimulation factors (e.g. oxidative stress) have been implicated in abnormal spermatogenesis and male infertility, however in many cases, the mechanisms causing abnormal spermatogenesis and male infertility are still evasive.

Testis is the location where spermatogenesis and secretion of semen occur. Spermatogenesis, which include process of spermatogonia stem cells proliferation or differentiation into spermatocytes, differentiation of spermatocytes, meiotic division, differenciation of round spermatids and release of mature spermatozoa, mainly take place in the seminiferous tubules. Straight seminiferous tubules are the first segment of sperm excretion, while leydig cells in the testicular interstitium secrete male hormones. Dysfunction in any of these parts and/or process could cause abnormal semen parameters and male infertility. In addition, the central nervous sex hormone secretion of the hypothalamic-pituitary–gonadal (HPG) axis plays an important role in the regulation of spermatogenesis [3]. By puberty, hypothalamic gonadotropin releasing hormone (GnRH) stimulates the secretion of pituitary gonadotropins, mainly follicle stimulating hormone (FSH) and luteinizing hormone (LH), which stimulate the testis to secrete T allowing for the differentiation of spermatogonial stem cells, and the spermatogonia continue to proliferate and ultimately develop into spermatozoa.

Renin-angiotensin system (RAS) is a hormone system that plays critical role in blood pressure control [4]. In recent decades, in-depth research on the RAS has revealed its new biological effects [5], and expression of those RAS factors have also been detected in many organs and tissues, suggesting the existence of localized RAS functions [6]. Recent studies have shown that renin-angiotensin system (RAS) may be involved in the regulation of male infertility and spermatogenesis [7]. A large number of gene and protein analyses have confirmed the presence of RAS components in the male reproductive system [8], including the testis, vas deferens, epididymis, prostate, sperm, and semen. The level of AngII in semen was found to be 3–5 times higher than that in the plasma [5].

The main biological effects of the RAS are largely dependent on AngII and through the receptor angiotensin receptor 1 (AT1) [9]. As documented, the two rat AT1 receptor isoforms (rAT1A and rAT1B) are pharmacologically indistinguishable from each other and from that of the human (hAT1) [10]. AT1 is expressed in male reproductive organs and central nervous system. It has been reported that the mRNA and protein expression of rAT1A and rAT1B could be detected in the testis, epididymis, and prostate tissues [11]. Immunohistochemistry showed the AT1 expression in the tail of mature spermatozoa and in the head and tail of ejaculated mouse spermatozoa [12]. In addition, Lin and co-workers used in situ hybridization to measure AngII mRNA in the brainstem and demonstrated a relationship between AT1a and AngII expression. AT1 could be detected in the brainstem and hypothalamus [13]. Moreover, In vitro experiments of human and animal spermatozoa further showed that AngII could stimulate sperm motility, increase the level of intracellular calcium in the spermatozoa through AT1, and induce the sperm acrosome reaction [12, 14].

Although the expression of AT1 receptor in central nervous system, hypothalamus and pituitary has been found for a long time [15, 16], whether it is involved in the regulation of GnRH-FSH-LH sex hormones, and whether it is involved in the regulation of spermatogenesis and male infirmity through this mechanism has not been clarified and need to be further explored.

The aim of this study is to detect the effects of RAS and AT1 on male spermatogenesis and reproductive ability by AT1 gene knockout and angiotensin receptor blocker (ARB) treatment.

## Materials and methods

### Animals model establishment

Male C57BL/6 mice and heterozygous ± AT1KO mice were provided by Shanghai Shrek Experimental Animal Co., Ltd (Shanghai). AT1KO heterozygous mice (± AT1KO) model was initially established by the National Natural Science Foundation of China to study the role of ± AT1KO in a chronic renal failure model on cardiac remodeling and to determine the potential curative effect of the traditional Chinese medicine Shenxinning recipe (30,873,262, 2009–2011). Experiment male ± AT1KO mice were selected from offspring of a mating between normal mice and female ± AT1KO mice. The animals were raised in the clean feeding room of the Experimental Animal Center of Shanghai University of Traditional Chinese Medicine. Experimental mice were divided into the following groups (n = 20 per group): Group A, normal C57BL/6 mice perfused with normal saline; Group B, normal C57BL/6 mice intragastrically administered with angiotensin receptor blocker (losartan potassium) (ARB group); Group C, male ± AT1KO mice perfused with normal saline. Ten mice per group were sampled at 2 weeks and 4 weeks, respectively. This study was approved by the Shanghai Animal Ethics Review Committee.

### Pregnancy test

Mice were caged at a ratio of 2 to 1 in males and females at week 2 and week 4 after grouping, and the pregnancy rate of female mice was determined in one week of cage closure (when vaginal plugs were detected, animals were considered to be pregnant).

### Sperm count and morphology analysis

The epididymis tissue was harvested, added to 1 ml phosphate-buffered saline, and ground. The tissue fragments were filtered, and the number of spermatozoa in the suspension was counted on a blood cell count plate under a microscope (Nikon E200, Tokyo, Japan). The suspension was placed on slides and fixed with formaldehyde for 5 min. After eosin staining for 1 h, spermatozoa of the mice were observed under a microscope (Nikon E200, Tokyo, Japan). The sperm morphology was assessed and the sperm deformity rate was calculated. Sperm deformity was identified by two experienced doctors from Reproductive Center separately. Compared with normal sperms, the sperms with fat head, folded tail, no hook, banana shape, amorphous, double head or double tail were counted as abnormal. Taleless sperms, head overlapping sperms or whole overlapping sperms were not counted. The Normal Rate of sperm was equal to (1- the number of malformed sperms / the number of totally counted sperms) × 100%. This assessment was performed at Reproductive Center of Dawning Hospital.

### Expression measurements

#### ELISA tests

The levels of the RAS components, including renin, ACE, ACE2, AngI, AngII, AngIII, and AngIV, were measured in the tissue homogenate with enzyme-linked immunoassay (ELISA, Shanghai Bogu Biotechnology Co., Ltd.). Elisa kit (Shanghai Bogu Biotechnology Co., Ltd.) was used to determine the concentrations of testosterone, GnRH, FSH and LH in the plasma of mice.

#### Quantitative real-time polymerase chain reaction (QRT-PCR)

Total RNA was extracted from the homogenate of tissues (testis, pituitary and hypothalamus of mice) with TRIzol reagent (Life Technologies). Reverse transcription was performed with oligo deoxythymidine (oligo-dT) primers and reverse transcriptase (Promega) according to the manufacturer's protocol. Primers were designed based on sequences in GenBank using Primer5.0 software (Table 1), and were synthesized by Shanghai Semitic Biotechnology Co., Ltd (Shanghai). Quantitative real-time PCR (RT-qPCR) assay was performed with GoTaq qPCR Master Mix (Promega) and an ABI 7300 Fast Real-Time PCR Detection System (Applied Biosystems). Briefly, a-20 μL PCR reaction that included 1 μL of complementary DNA, 10 μL of GoTaq qPCR Master Mix, and 0.2 μM of each primer was used and adjusted to the final volume with double distilled H2O (ddH2O). mGAPDH in parallel for each run was used as an internal control. The reactions were set up on the basis of the manufacturer's protocol. PCR conditions were incubation at 95 °C for 3 min followed by 40 cycles of thermal cycling (10 s at 95 °C, 20 s at 58 °C, and 10 s at 72 °C). The relative expression ratio of mRNA was quantified via the 2(ΔΔCt) method.

**Table 1:** Primer sequence

Gene	Primer	Synthesized sequence
mAT1	Forward Primer	TTCATTGAGAACACCAATATCACTG
	Reverse Primer	GCTGGTAAGAATGATTAGGAAAGG
	Probe	CGAGTCCCGGAATTCAACGCTCC
AngII	Forward Primer	CGGAACGACCTCCTGACTTG
	Reverse Primer	GCAGGTCCTGCAGATTGTAGG
	Probe	CCGCCTGACTCTGCCCCAGC
mGAPDH	Forward Primer	TGGAGTCTACTGGCGTCTT
	Reverse Primer	TGTCATATTTCTCGTGGTTCA
	Probe	CTGAAGGGTGGGGCCAAAAG

### Statistical analysis

The numerical data are expressed as the mean and standard deviation. Means between two groups were compared by Student’s t-test, and means of multiple groups were compared by one-way analysis of variance. Data with non-homogeneous variance were compared with a non-parametric test. Categorical data was expressed as percentage and the differences between groups were analyzed using Chi-square test. All statistical analyses were performed with SPSS13.0 software. *P* < 0.05 and *P* < 0.01 was considered significant and very significant difference, respectively.

## Results

### AT1 expression in male ± AT1KO mice

**Fig. 1:**
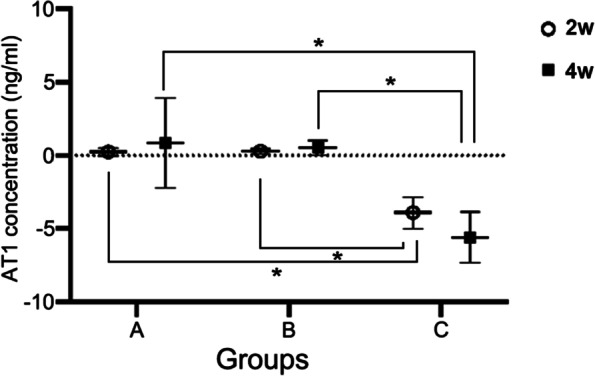
The changes of AT1 concentrations in the testes of normal C57BL/6 mice (**A**), RAB mice (**B**), and ± AT1KO mice (**C**) during the second (○) and fourth weeks (□). Error bar indicates the mean ± SD and significances are indicated as ∗ *p* < 0.01

AT1 expression was detected in the testis, pituitary and hypothalamus of mice in Control, ARB and ± AT1KO groups by QRT-PCR. Compared with normal mice, the AT1 expression levels in all three tissues of ± AT1KO mice were significantly decreased (*P* < 0.01) while there was no difference between Control and ARB groups (Table 2). The protein expression levels of AT1 in the testes of mice in Control, ARB and ± AT1KO groups were detected using ELISA method during the second and fourth weeks after grouping. Compared with mice in Control and ARB groups, AT1 expression in the testes of ± AT1KO group was significantly decreased (*P* < 0.01) (Fig. 1).

**Table 2:** The mRNA expression of AT1 in the testis, pituitary and hypothalamus of mice in Control, ARB and ± AT1KO groups

AT1		Control	ARB	± AT1KO
Testis	W2 (△Ct)	10.24 ± 0.29	10.29 ± 0.16	7.91 ± 1.07**
	W4 (△Ct)	10.85 ± 3.09	10.53 ± 0.50	5.60 ± 1.75**
Pituitary	W2 (△Ct)	14.94 ± 0.21	14.08 ± 1.10	7.36 ± 0.69**
	W4 (△Ct)	14.61 ± 1.72	14.81 ± 1.41	7.72 ± 0.10**
Hypothalamus	W2 (△Ct)	14.02 ± 1.58	14.53 ± 1.11	7.47 ± 0.94**
	W4 (△Ct)	13.27 ± 0.48	13.02 ± 1.00	7.86 ± 1.15**

^**^P < 0.01 compared to control or ARB groups

### AT1 partial knockout and ARB did not affect Ang levels

The expression levels of Ang, including AngI, AngII, AngIII, and AngIV, were measured in the plasma with ELISA and RNA expression levels of total Ang in the tissues (the testis, pituitary and hypothalamus) were detected with QRT-PCR. The results showed that there was no significant difference of Ang levels among Control, ARB and ± AT1KO groups (Table 3).

**Table 3:** Ang expression in Control, ARB and ± AT1KO groups

Plasma Ang		Control	ARB	AT1-KO
AngI	W2 (ng/ml)	5.12 ± 0.60	5.16 ± 0.43	5.77 ± 1.33
	W4 (ng/ml)	4.16 ± .68	5.00 ± 0.91	4.39 ± 0.53
AngII	W2 (ng/ml)	5.24 ± 2.28	5.71 ± 2.12	4.17 ± 1.27
	W4 (ng/ml)	5.53 ± 1.16	5.25 ± 1.73	4.59 ± 1.22
AngIII	W2 (ng/ml)	650.72 ± 91.08	599.56 ± 10.15	607.00 ± 48.35
	W4 (ng/ml)	617.09 ± 58.25	617.21 ± 71.54	624.93 ± 45.33
AngIV	W2 (ng/ml)	21.07 ± 8.75	36.64 ± 11.09	30.08 ± 59.49
	W4 (ng/ml)	28.15 ± 6.65	38.00 ± 13.75	25.83 ± 19.70
Total Ang mRNA		Control	ARB	AT1-KO
Testis	W2 (△Ct)	4.43 ± 1.02	5.39 ± 0.65	5.35 ± 2.07
	W4 (△Ct)	6.25 ± 3.55	5.75 ± 0.50	4.77 ± 1.47
Pituitary	W2 (△Ct)	2.48 ± 0.44	1.83 ± 1.41	2.96 ± 0.73
	W4 (△Ct)	2.86 ± 1.69	1.94 ± 0.81	2.1 ± 0.18
Hypothalamus	W2 (△Ct)	0.83 ± 0.53	1.91 ± 0.54	1.98 ± 1.17
	W4 (△Ct)	1.75 ± 0.52	1.25 ± 1.21	2.31 ± 1.05

### AT1 knockout reduced GnRH level but not FSH or LH

ELISA was used to determine the concentrations of GnRH, FSH, and LH in plasma of mice in Control, ARB and ± AT1KO groups. And the results showed that the GnRH level (*P* < 0.01) but not FSH or LH was decreased in ± AT1KO group (Table 4).

**Table 4:** The expression levels of GnRH, FSH and LH in plasma of mice in Control, ARB and ± AT1KO groups

		Control	ARB	AT1-KO
GnRH	W2 (ng/ml)	343.76 ± 27.04	312.25 ± 39.61	128.15 ± 46.10**
	W4 (ng/ml)	304.99 ± 37.88	190.72 ± 21.33	148.84 ± 44.44**
FSH	W2 (ng/ml)	3.32 ± .92	2.99 ± 1.51	2.41 ± 1.14
	W4 (ng/ml)	3.51 ± 1.54	3.13 ± 1.09	4.07 ± 1.13
LH	W2 (ng/ml)	10.00 ± 2.08	9.19 ± 1.66	11.62 ± 3.42
	W4 (ng/ml)	14.04 ± 4.75	9.15 ± 4.77	16.52 ± 5.56

^**^P < 0.01 compared to control or ARB groups

### The male infertility identification of ± AT1KO mice

#### Pregnancy rate of the female mice mated with ± AT1KO male mice

The pregnancy rate of female mice was determined in one week of cage closure with male mice in Control and ± AT1KO groups. None of the female mice mated with ± AT1KO male mice became pregnant. Moreover, the pregnancy rate in 2-week normal mice was 50%, and the pregnancy rate in 4-week normal mice was 55% (Table 5). The plasma testosterone levels were detected in these mice but no significant difference was found (data not shown).

**Table 5:** Pregnancy rate of the female mice mated with ± AT1KO male mice

Group	Pregnancy rate (%) (2 W)	Pregnancy rate (%) (4 W)
Control	50%	55%
± AT1KO	0	0

#### Teratozoospermia of ± AT1KO mice

**Fig. 2:**
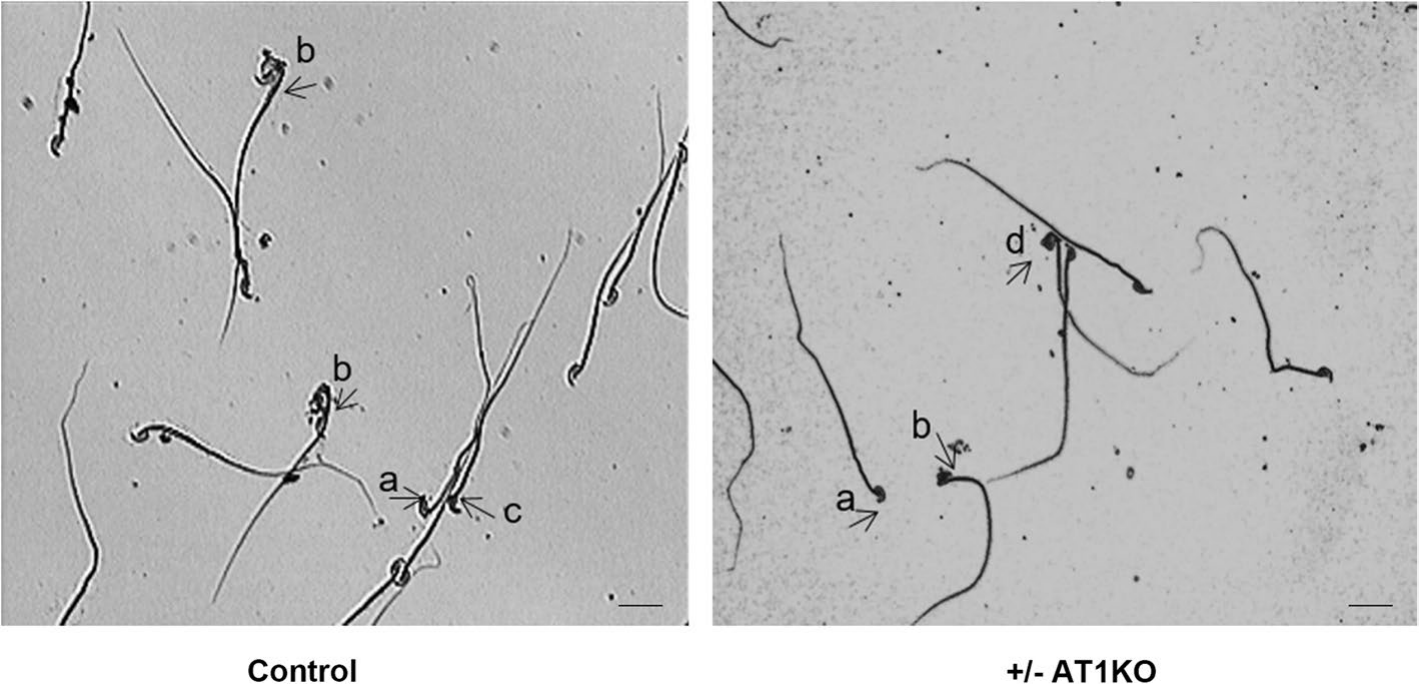
The sperm morphology of normal mice and ± AT1KO mice. Note: a. no hook b. amorphous c. banana head d. fat head. Scale = 25 μm

Deformation rate of spermatozoa in Control, ARB and ± AT1KO groups were assessed. The main deformities observed in mice from all three groups included large (fat) or abnormal (banana) shaped head, folded tail, no hook, and indefinite (amorphous) form, while no double head or double tail were detected (Fig. 2). The total deformity rate was 59% in ± AT1KO mice, significantly (*P* < 0.01) higher than those in normal mice (19%) and in ARB mice (25%). Enlarged (fat) head appeared to be the mostly increased deformity in spermatozoa from ± AT1KO mice (201/642, compared to 26/990 in control group and 23/708 in ARB group). Spermatozoa with no hook and folded tails were also significantly increased. On the other hand, sperms with indefinite form only slightly increased while the rate of sperms with banana shaped were decreased compared to the control and ARB groups (Table 6).

**Table 6:** Analysis of sperm morphology in Control, ARB and ± AT1KO mice groups

Group	The number of totally counted sperms^a^	The number of malformed sperms^a^							Normal rate^b^	Deformity rate^b^
		No hook	Indefinite form	Banana head	Fat head	Double tail	Double head	Tail folding		
Control	990	21	61	60	26	0	0	20	81%	19%
ARB	708	12	69	52	23	0	0	21	75%	25%
± AT1KO	642	43	78	22	201	0	0	35	41%**	59%**

^**^P < 0.01 compared to control or ARB groups

^a^The number of totally counted and malformed sperms in 10 mice per group. The median numbers are presented

^b^Deformity rate = the number of malformed sperms/ the number of totally counted sperms*100%; Normal rate = (1- Deformity rate)%

## Discussion

In this study, we demonstrated the decreased AT1 expression in ± AT1KO mice testes, which resulted in reduced GnRH level. These results indicated that AT1 may play role in the male infertility by contributing teratozoospermia in testis. In addition, the reduced GnRH levels in ± AT1KO animals indicate the function of AT1 in spermatogenesis may be related with HPG axis.

AngII is the main effector hormone of the RAS in the male reproductive system, and it exerts its function by binding to specific receptors (AT1 and AT2). The RAS mainly carries out its physiological effects through AngII and regulates sperm function [7]. However, local RAS has been reported to have distinct effects in various tissues from those observed for circulating RAS [17]. Large amounts of various RAS components (ie, renin, angiotensinogen, Ang I, Ang II, and angiotensin-converting enzyme) have also been found in the pituitary, adrenal gland, and male and female reproductive systems [18], suggesting that RAS may play an important role in the reproductive activity of the whole body or local tissues through circulating, paracrine, or autocrine effects. Local RAS activity has been detected in many parts of the male reproductive system such as the testis, epididymis, and prostate, suggesting an association with male reproduction [8]. These potential functions need to be further explored.

Recently, some researchers have indicated AngII could bind to AT1 to regulate various physiological processes such as vasoconstriction, aldosterone release, humoral regulation, and cell growth [14, 19, 20]. Most studies have focused on the effects of AngII on testicular Leydig cells [21], sperm motility [22], acrosome reaction [23], and related processes. However, these mechanisms and effects require further validation. Moreover, a series of studies have proven that AT1 plays a regulatory role in male reproduction [24]. However, in this study, only a small amount of AT1 was detected in the testes of ± AT1KO mice. And it was found in the observation of sperm morphology and fertility that none of the ± AT1KO mice successfully impregnated a female and the ± AT1KO mice also exhibited clear sperm morphology abnormalities. Overall, these results showed that antagonizing the RAS especially the knockout of AT1 receptor could significantly affect the reproductive function of male mice.

Previous study have indicated that HPG axis and GnRH-FSH-LH gonadotropin axis could regulate RAS system [25], while the Ang reverse regulation for the hormone axis is rarely studied. Moreover, Ang/AT1 seems to be unable to regulate FSH or LH [26], but the function of Ang in regulating GnRH is still unclear. In this study, our research results showed that AT1 knockout and ARB seemed to cause the downregulation of GnRH (not statistically significant in ARB group, but there was a trend), but this effect did not affect the downstream FSH and LH levels. Considering the expression of GnRH receptor (GnRH-R) in testis [27], GnRH downregulation after the knockout of AT1 receptor may play a role in production of abnormal sperm through other signaling pathways. As reported, two forms of GnRH (I and II) and two types of receptors (GnRHR-I and -II) are present in the male reproductive system of the human and non-human primate, playing an essential role in the control of reproduction while operating primarily at the HPG axis [28]. However, GnRH may still have direct effects on the testis and lead to sperm anomalies without regulating FSH or LH, which needs further research. The role of Ang in the process of spermatogenesis and its relationship with gonadotropin expression needs further research. And it is necessary to study the effect of AT1 receptor on GnRH levels in neurons and tissues directly to further study the role of Ang in regulating spermatogenesis.

Since the main biological effects of the RAS are largely dependent on AngII and realized through the receptor AT1 [20], knocking out AT1 is an effective method to antagonize the RAS. To further identify the cause of infertility in ± AT1KO male mice, we detected AT1 expression in the testis to explore whether AT1 is associated with teratozoospermia in testis, which most strongly affects the reproductive function of mice. And a small amount of AT1 was detected in the testes of ± AT1KO mice. Nevertheless, the lack of pregnancy from the ± AT1KO mice suggested that the RAS could affect reproduction through AT1 receptors, but that this does not involve the action of AT2 receptor. Since the signal transduction pathway of AT2 is not clear, it needs to be further explored, whether the influence of the RAS on the reproductive system is mediated by AT2.

There are some limitations in this study. mRNA levels of AT1 receptor in the testis, pituitary and hypothalamus of male ± AT1KO mice were detected in this study but we only detect the protein expression levels of AT1 in testes using ELISA. The male infertility of ± AT1KO mice was assessed by pregnancy rate of the female mice mated with ± AT1KO male mice and the teratozoospermia of ± AT1KO mice. However, due to the limitation of experimental conditions, investigation of sperm movement patterns and measurement of sperm relative velocities cannot be fulfilled. Further studies are needed in the future. Moreover, the function of Ang/AT1 in regulating GnRH without affecting the downstream FSH and LH levels, indicating that the GnRH may have direct effects on the testis leading to the sperm anomalies observed, is still unclear.

## Conclusions

In summary, we demonstrated the decreased AT1 expression in ± AT1KO mice testes. And our research results indicated AT1 may contribute teratozoospermia in testis, and provided a new target for male infertility treatment.

## Data Availability

The datasets used and/or analysed during the current study are available from the corresponding author on reasonable request.

## Abbreviations

AT1: Angiotensin receptor 1
KO: Knock out
HPG: Hypothalamic-pituitary–gonadal
QRT-PCR: Quantitative real-time polymerase chain reaction
ARB: Angiotensin receptor blocker
FSH: Follicle stimulating hormone
LH: Luteinizing hormone
GnRH: Gonadotropin releasing hormone
RAS: Renin-angiotensin system
ABI: ABI7300 fluorescence quantitative PCR instrument
